# Analytical and Clinical Performance of VIDAS^®^ HIV DUO AG/AB Assay for Enhanced Early HIV Diagnosis

**DOI:** 10.3390/diagnostics16182954

**Published:** 2026-09-12

**Authors:** Véronique Lemée, Mathilde Lacôte, Peggy Nomade, Yves Mérieux, Sandrine Gréaume, Isabelle Voisin, Laurence Bridon, Jean-Christophe Plantier, Elodie Alessandri-Gradt, Vinca Icard

**Affiliations:** 1 Univ Rouen Normandie, Université de Caen Normandie, INSERM, Normandie Univ, DYNAMICURE UMR 1311, CHU Rouen, Department of Virology, F-76000 Rouen, France; veronique.lemee@chu-rouen.fr (V.L.); elodie.alessandri@chu-rouen.fr (E.A.-G.); 2 bioMérieux, 69280 Marcy l’Etoile, France; mathilde.lacote@biomerieux.com (M.L.); laurence.bridon@biomerieux.com (L.B.); 3 Etablissement Français du Sang (EFS) Auvergne-Rhône-Alpes (AURA), 69150 Décines, France; yves.merieux@efs.sante.fr; 4 Etablissement Français du Sang (EFS) Hauts de France—Normandie (HFNO), 76000 Rouen, France; sandrine.greaume@efs.sante.fr (S.G.); isabelle.voisin@efs.sante.fr (I.V.); 5 Univ Rouen Normandie, Université de Caen Normandie, INSERM, Normandie Univ, DYNAMICURE UMR 1311, CHU Rouen, Department of Genomic Microbiology, F-76000 Rouen, France; jc.plantier@chu-rouen.fr; 6 Croix Rousse Hospital, Hospices Civils de Lyon (HCL), Laboratory of Virology, 69004 Lyon, France; vinca.icard@chu-lyon.fr

**Keywords:** HIV, diagnostic, serology, antigen, p24, acute infection, fourth generation, sensitivity, specificity

## Abstract

**Background/Objectives**: Early detection of HIV infection is critical for timely clinical management and prevention of transmission. Fourth-generation antigen/antibody assays improve early diagnosis by detecting p24 antigen prior to seroconversion. This study evaluated the analytical and clinical performance of the VIDAS^®^ HIV DUO AG/AB assay. **Methods**: A multicenter evaluation was conducted using WHO HIV-1 p24 standards, 50 HIV-1/HIV-2 culture supernatants representing diverse genotypes, 40 seroconversion panels, 634 HIV-positive clinical specimens, 6063 HIV-negative samples from four populations, and 272 potentially cross-reactive samples. Precision was assessed according to CLSI EP05-A3 guidelines. Performance was evaluated against VIDAS^®^ HIV DUO Ultra and contextualized with results reported for other fourth-generation assays. **Results**: VIDAS^®^ HIV DUO AG/AB showed a lower p24 antigen limit of detection (0.3 IU/mL) than VIDAS^®^ HIV DUO Ultra (0.5–0.6 IU/mL) and higher relative p24 reactivity across multiple genotypes. It enabled earlier detection in more than 30% of seroconversion panels, corresponding to a cumulative gain of 60 days versus VIDAS^®^ HIV DUO Ultra. Based on historical datasets, gains of up to 29 days were observed relative to reported performances of the ARCHITECT HIV Ag/Ab Combo and MAGLUMI HIV Ab/Ag Combi assays. All 634 HIV-positive clinical samples were detected, demonstrating broad genotype inclusivity within the diversity of HIV strains represented in this study. Specificity reached 99.95% (95% CI: 99.90–99.99%), with no cross-reactivity observed. Precision coefficients of variation were below 3.5%. **Conclusions**: The VIDAS^®^ HIV DUO AG/AB assay demonstrated high analytical sensitivity, early detection in seroconversion panels, broad genotype coverage, and excellent (>99.5%) specificity, supporting its use as a fourth-generation option for HIV screening in various clinical and epidemiological settings.

## 1. Introduction

More than 40 years after its emergence, HIV/AIDS remains one of the world’s greatest health challenges. Despite substantial progress in treatment and prevention, the epidemic continues to affect millions of people worldwide. As of 2023, approximately 40 million people were still living with HIV-1 globally, with about 1.5 million new cases and 630,000 deaths reported that year [1,2].

Available antiretroviral treatments can control the replication of the virus and maintain an undetectable viral load, significantly reducing HIV-related mortality and morbidity [3]. However, there is still no definitive vaccine or curative treatment [4]. For about ten years, HIV-negative individuals have had access to a prophylactic drug treatment (PrEP, pre-exposure prophylaxis) that combines two antiretrovirals and effectively reduces virus transmission. The protection is over 90% but is not completely effective [5,6,7]. Therefore, prevention remains essential and includes regular testing to determine serological status [8]. The earlier the virus is detected, the more effective the treatment can be, thus improving the quality of life and life expectancy of infected individuals. Additionally, knowing one’s serological status helps prevent the transmission of the virus to others. Early testing also enables rapid access to antiretroviral treatments that control the viral load, making it undetectable and thus limiting damage to the immune system.

HIV-1 acute phase infection corresponds to the first weeks following contamination, generally between 2 and 6 weeks. During this period, the virus replicates rapidly, and the viral load reaches very high levels, increasing the risk of transmission [9,10,11]. Consequently, early detection of acute infection is crucial. During the acute phase, the p24 antigen, a protein component of the virus capsid, can be detected before the appearance of antibodies, which occurs later, generally 2 to 4 weeks after the onset of infection [12]. Combined tests (p24 antigen and anti-HIV antibodies) thus shorten the serological window during which anti-HIV antibodies are not yet detectable by antibody-only tests. Minimizing this serological window is critical, and ideally, the assay should detect the HIV-1-specific p24 antigen at the lowest possible limit of detection [13]. Within this context, fourth-generation HIV immunoassays have been developed to reduce the window of detection during the seroconversion period by 4–8 days, and assays such as VIDAS^®^ HIV DUO Ultra and VIDAS^®^ HIV DUO Quick (bioMérieux, France) have long been used on the VIDAS^®^ platform for HIV screening. These automated assays enable simultaneous detection of HIV-1 p24 antigen and anti-HIV-1/2 antibodies and, depending on the assay configuration, may report these two responses separately. Their design allows earlier identification of primary infections by integrating antigen detection prior to seroconversion [14,15], while maintaining reliable antibody detection once seroconversion has occurred. These fourth-generation tests have very high sensitivity, often exceeding 99%, with equally high specificity [16,17].

A new assay, VIDAS^®^ HIV DUO AG/AB, has now been developed to replace both VIDAS^®^ HIV DUO Ultra and VIDAS^®^ HIV DUO Quick. This new-generation test preserves the dual Ag/Ab detection capability with antigen and anti-HIV differentiation, while aiming to further improve early detection performance, analytical sensitivity, specificity and overall robustness. Given the rapid evolution of HIV diagnostics and the emergence of other highly sensitive fourth-generation tests—including ARCHITECT HIV Ag/Ab Combo [18,19] and other advanced combination assays [20,21] with reported sensitivities approaching 100% and very high specificities—a comparative performance evaluation is essential. The objective of this study is therefore to compare the diagnostic performance of the new VIDAS^®^ HIV DUO AG/AB assay with existing fourth-generation HIV screening tests across different analytical platforms—including the currently marketed VIDAS^®^ HIV DUO Ultra—with special emphasis on early infection detection, sensitivity, specificity, and p24 antigen detection limits, which are critical parameters for reducing the diagnostic window.

## 2. Materials and Methods

### 2.1. Study Design

**Figure 1:**
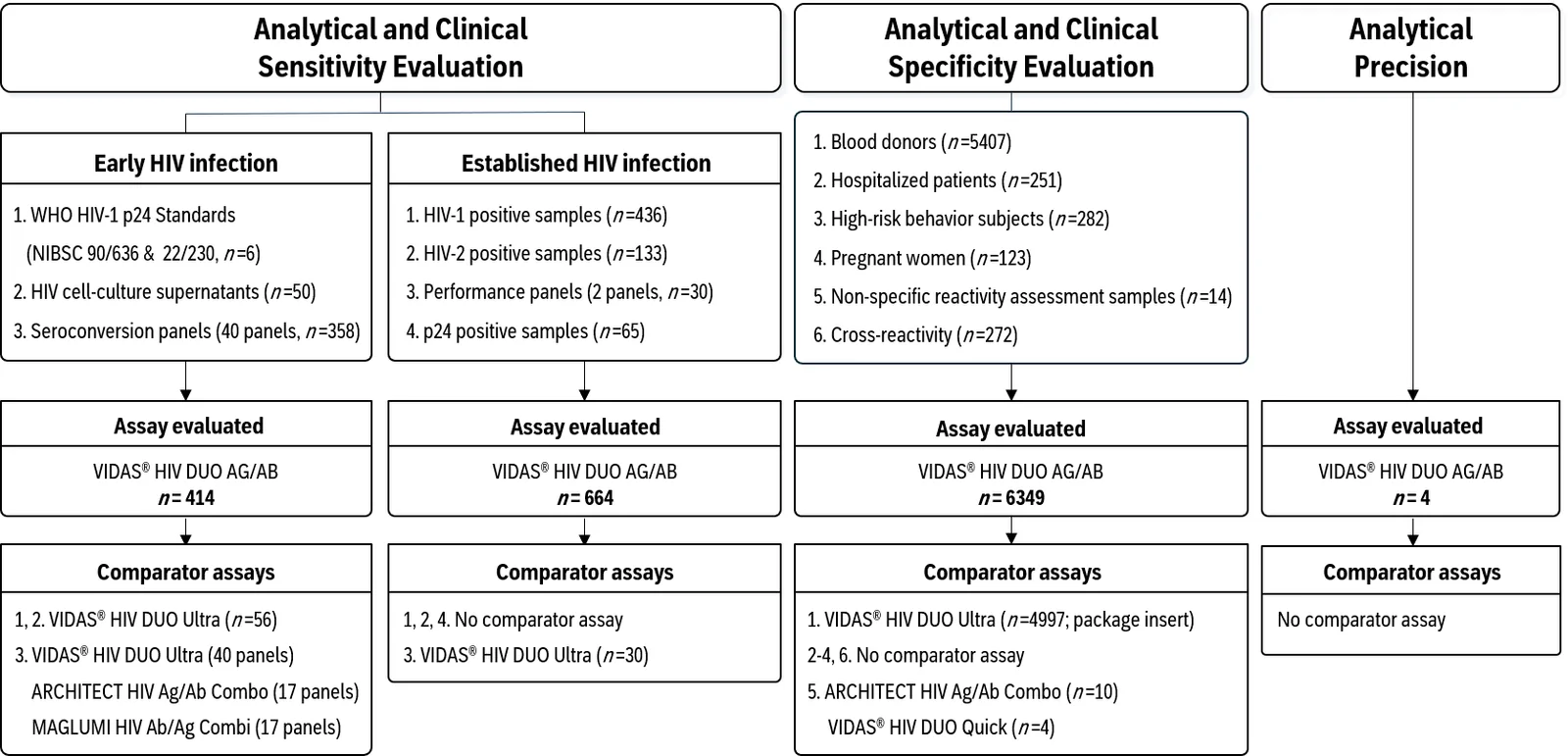
Study workflow and sample distribution, with sample numbers (*n*) in brackets. Comparative data for ARCHITECT HIV Ag/Ab Combo and MAGLUMI HIV Ab/Ag Combi in the sensitivity evaluation were obtained from Wang et al. (2024) [22].

This multicenter study combined retrospective and prospective sample collections to evaluate the analytical and clinical performance of the VIDAS^®^ HIV DUO AG/AB. As outlined in Figure 1, the evaluation included four complementary analyses: (i) analytical and clinical sensitivity in early HIV infection, (ii) clinical sensitivity in established HIV infection, (iii) analytical and clinical specificity, and (iv) analytical precision. Samples were collected from multiple clinical sites, blood establishments, and commercial suppliers according to the planned analyses (Figure 1).

### 2.2. Sample Collection

#### 2.2.1. Sensitivity Evaluation in Early HIV Infection

Three antigen panels were used to determine analytical sensitivity for the detection of HIV-1 antigen: (i) the WHO International Standard HIV-1 p24 Antigen (NIBSC 90/636); (ii) the 1st WHO International Standard for HIV-1 p24 Antigen (NIBSC 22/230); and (iii) a panel of 50 HIV cell-culture supernatants. The latter consisted of primary isolates representing a broad range of HIV diversity, including HIV-1 group M subtypes (*n* = 30), circulating recombinant forms (*n* = 10), HIV-1 group O (*n* = 6), and HIV-2 groups A and B (*n* = 4). Among these 50 supernatants, 45 were provided by the National Reference HIV Laboratory (Rouen, France)—a collection officially registered with the French Ministry of Research (declaration number DC 2023-5770). In addition, 3 cell-culture supernatants were obtained from BEI Resources-NIH HIV Reagent Program (Manassas, VA, USA), and 2 were sourced from bioMérieux (Marcy l’Étoile, France).

Forty seroconversion panels acquired from LGC Clinical Diagnostics Inc./Seracare Life Sciences (Milford, MA, USA) and Zeptometrix (Buffalo, NY, USA) were evaluated to assess assay diagnostic sensitivity during early seroconversion.

#### 2.2.2. Sensitivity Evaluation in Established HIV Infection

A total of 634 specimens from individuals with confirmed HIV infection were included in the diagnostic sensitivity assessment, comprising 569 leftover clinical specimens positive for HIV-1 or HIV-2 antibodies and 65 native HIV-1 p24 antigen-positive samples. The HIV screening workflow implemented at the Croix-Rousse Hospital (Lyon, France) is summarized in Supplemental Figure S1 (Algorithm for HIV infection screening). Most specimens were provided by the Croix-Rousse Hospital (Lyon, France), while additional samples originated from the Rouen University Hospital (Rouen, France) and from an internal bioMérieux collection (Marcy L’Etoile, France). The panel consisted of both retrospectively and prospectively collected samples.

Most retrospective samples included in the sensitivity analysis were obtained from the biological resource center (CRB) of the Hospices Civils de Lyon (HCL, Lyon, France). All patients were informed of the storage and potential research use of their biological material and associated data and provided written informed consent in accordance with French regulations. The CRB biobank is registered with the French Ministry of Research under authorization AC2008-73, with renewals AC2013-1867 and AC2019-3465.

Prospective samples included specimens collected during routine clinical activity, notably from free and anonymous testing centers, where participants were informed of the potential research use of their remaining samples in accordance with local procedures.

Of the total sample set, 30 specimens were freshly collected (<24 h) prior to testing. Clinical demographic information (age and gender) was available for 547 of the 569 individuals. Among these, 38.0% (*n* = 216) were reported as female and 58.2% (*n* = 331) as male, with ages ranging from 18 to 88 years.

The panel comprised 436 HIV-1-positive and 133 HIV-2-positive samples, representing a broad range of viral genetic diversity. The HIV-1 positive samples included multiple group M subtypes (A, A1, A2, B, C, D, F, F1, F2, G, J, K), circulating recombinant forms (CRF01_AE, CRF02_AG, CRF06_cpx, CRF11_cpx, CRF12_BF, CRF13_cpx, CRF14_BG, CRF18_cpx, CRF19_cpx, CRF22_01A1, CRF36_cpx, CRF44_BF, CRF45_cpx), as well as HIV-1 group O and unknown genotypes. A total of 134 HIV-1-positive and 48 HIV-2 positive individuals were receiving antiretroviral treatment at the time of sampling. The HIV-1-positive cohort also included 20 pregnant women, of whom 8 were multigravida and 12 were primigravida.

The diagnostic sensitivity assessment also included 65 native p24-positive samples, originating from multiple sources: 17 from an internal bioMérieux collection (Marcy L'Etoile, France), 39 from commercial performance panels provided by LGC Clinical Diagnostics Inc./Seracare Life Sciences (Milford, MA, USA), 1 specimen from the EFS of Centre Atlantique Tours (Tours, France), and 8 specimens from the Croix-Rousse Hospital (Lyon, France).

Two commercial antibody performance panels were used to assess antibody detection: the DQ121 panel from the EFS (Lille, France) (*n* = 20) and the AccuSet HIV-1 Early Infection Panel 0800-0394 from LGC Clinical Diagnostics Inc./Seracare Life Sciences (Milford, MA, USA) (*n* = 10), for a total of 30 samples.

#### 2.2.3. Specificity Evaluation

1. Blood donors

For the VIDAS^®^ HIV DUO AG/AB evaluation, specimens included 2842 blood donors from the Hauts-de-France—Normandie French Blood Establishment (EFS) and 2565 blood donors from the Auvergne-Rhône-Alpes EFS.

Blood donors were informed at each donation that their biological specimens might be used for research purposes, and only samples from donors who provided consent were included in this study. All donor data were anonymized, and no individual-level information was collected. Each participating EFS site supplied aggregated demographic information and certified that all included specimens were confirmed HIV-negative by both serological testing (Elecsys HIV Duo, Roche Diagnostics, Basel, Switzerland; and, when reactive, ARCHITECT HIV Ag/Ab Combo, Abbott Diagnostics, Abbott Park, IL, USA) and viral genome detection (Procleix^®^ Tigris^®^ System and Procleix^®^ Ultrio^®^ Assay (Grifols Diagnostic Solutions Inc., Emeryville, CA, USA)). In accordance with French regulations, no additional consent or non-opposition form was required for the use of these anonymized data. The HIV screening workflow applied at the QBD (Qualification Biologique du Don) sites of the French Blood Establishments is summarized in Supplemental Figure S2.

For the specificity evaluation of the VIDAS^®^ HIV DUO Ultra assay (bioMérieux, Marcy L'Etoile, France), a total of 4997 blood-donor specimens were included. These samples were collected prospectively from three regional divisions of the EFS. The dataset comprised 1498 specimens from the Auvergne–Rhône-Alpes region, 997 specimens from the Normandie region, and 2502 specimens from the Nord–Pas-de-Calais region. These specimens were historically collected as part of the clinical evaluation of the VIDAS^®^ HIV DUO Ultra assay, and the corresponding data originated from the assay’s package insert.

2. Hospitalized patients, high-risk individuals, and pregnant women

This part of the specificity analysis included specimens recruited from the Croix-Rousse Hospital (Lyon, France), comprising 251 hospitalized patients from various clinical units and 282 individuals with behaviors associated with an increased risk of HIV acquisition. This latter group included 207 people attending the CeGIDD sexual health center and 75 individuals receiving pre-exposure prophylaxis (PrEP; Truvada).

The study population also incorporated a dedicated subgroup of 123 pregnant women recruited from two clinical sites: 50 specimens from the Saint-Luc—Saint-Joseph Hospital (Lyon, France) and 73 specimens from the Obstetrics department of the Croix-Rousse Hospital (Lyon, France).

Prospectively collected participants from the Croix-Rousse Hospital were informed of the potential research use of their remaining samples either through on-site written and oral information or by postal notification allowing one month for objection. At Saint-Luc—Saint-Joseph Hospital, participants were informed through an information note, and leftover samples were used in accordance with the French non-opposition procedure.

3. Non-specific reactivity assessment samples

A total of 14 HIV-negative specimens corresponding to false-positive results obtained with comparator assays were used for the non-specific reactivity assessment. These samples included 10 specimens initially reactive with the ARCHITECT HIV Ag/Ab Combo assay and 4 specimens initially reactive with the VIDAS^®^ HIV DUO Quick assay. All samples were provided by the Rouen University Hospital (CHU de Rouen, Rouen, France). These leftover specimens were collected as part of routine clinical activity, subsequently anonymized before testing, and confirmed as HIV-negative according to the local diagnostic algorithm before inclusion in the study.

4. Cross-reactive samples

A large panel of 272 potentially cross-reactive samples (218 serum samples and 54 plasma samples), representing 19 different categories, was obtained from commercial suppliers, the Saint-Joseph Saint-Luc Hospital, and the Centre Atlantique EFS. The panel included samples from conditions frequently associated with IgM responses (e.g., HAV, HBV, HCV, HEV, HSV, EBV, CMV, rubella, toxoplasmosis, and syphilis) to evaluate potential interference during acute infections. At Saint-Luc—Saint-Joseph Hospital, participants were informed through an information note, and leftover samples were used in accordance with the French non-opposition procedure. For samples originating from the EFS, only specimens from donors who had provided consent for research use were included.

#### 2.2.4. Precision Evaluation

Four samples were prepared to support the precision study. For the antibody response, two plasma pools (low and high levels) were generated by spiking defibrinated HIV-negative plasma—provided by Grifols Bio Supplies (Los Angeles, CA, USA)—with two commercially sourced HIV-positive specimens. For the antigen response, two additional plasma pools (low and high levels) were obtained by spiking defibrinated HIV-negative plasma with HIV-1 cell-culture supernatants corresponding to subtypes C and D, which were provided by the National Reference HIV Laboratory (Rouen, France).

### 2.3. HIV Assays

The VIDAS^®^ HIV DUO AG/AB assay, as well as the other fourth-generation serological assays and the viral genome detection methods used in this study, were performed in accordance with the manufacturers’ instructions.

The VIDAS^®^ HIV DUO AG/AB assay is an automated, qualitative, fourth-generation enzyme immunoassay performed on the VIDAS^®^ family instruments (bioMérieux, Marcy L'Etoile, France). The assay is based on Enzyme Linked Fluorescent Assay (ELFA) technology and combines, within a single automated test, two independent immunoassays that allow the differentiated detection of HIV-1 p24 antigen (AG response) and total antibodies directed against HIV-1 (groups M and O) and HIV-2 (AB response).

The assay uses a Solid Phase Receptacle (SPR), which serves both as the solid phase and the pipetting device. The upper part of the SPR is coated with a cocktail of monoclonal antibodies for the detection of HIV-1 p24 antigen. The lower part is coated with HIV-specific antigens, including recombinant full-length HIV-1 group M gp160 and a synthetic peptide from the immunodominant regions of HIV-1 group O gp41 and HIV-2 gp36, enabling the capture of anti-HIV antibodies.

The assay is performed in a disposable reagent strip containing all reagents required for sample processing, washing, immune-complex formation, and fluorescence detection. The analytical sequence, including reagent dispensing, incubation, washing, and signal measurement, is fully automated by the VIDAS^®^ family instrument.

During the assay, the sample and biotinylated detection reagents are cycled in and out of the SPR. HIV-1 p24 antigen released from viral particles is captured by anti-p24 monoclonal antibodies coated on the upper part of the SPR and detected through a sandwich immunoassay using biotinylated anti-p24 antibodies. Simultaneously, anti-HIV-1/2 antibodies bind to the HIV antigens coated on the lower part of the SPR and are detected by biotinylated HIV antigens using a double-antigen sandwich format. After washing to remove unbound material, a second incubation is performed in the antibody-detection zone with biotinylated antigens and peptides to promote double-antigen sandwich formation and reduce the risk of a hook effect. Following an additional wash step, a third incubation is carried out with an alkaline phosphatase-labeled streptavidin conjugate which binds to the biotinylated detection reagents associated with either the antigen or antibody immune complexes. After removal of excess conjugate by washing, addition of the fluorescent substrate 4-methylumbelliferyl phosphate generates a fluorescent signal proportional to the amount of immune complex formed. Fluorescence is measured separately for the antibody and antigen reactions, and the instrument automatically converts the signals into AG and AB index values and calculates signal-to-cutoff (S/CO) ratios for each response using stored calibration data.

The final test interpretation is considered reactive when either S/CO value is ≥1.00 for either response, regardless of the status of the other. A non-reactive interpretation is given when both antigen and antibody S/CO values are below 1.00.

In certain situations, extremely high concentrations of one HIV response (antibodies or antigen) may prevent accurate index calculation for the other response. In such cases, the affected response is reported as ND (“Not Determinable”) and is referred to as a masked response, while the response for which a valid index can be calculated constitutes the unmasked response. The final interpretation relies exclusively on the unmasked response and remains valid; a sample is considered reactive if the unmasked S/CO is ≥1.00.

Conversely, in some negative specimens, very low antigen signals may also result in an ND antigen index; when the antibody index is simultaneously below the cutoff, the sample is interpreted as negative.

### 2.4. Methods

#### 2.4.1. Sensitivity Evaluation in Early HIV Infection

The VIDAS^®^ HIV DUO Ultra assay, the predecessor of the VIDAS^®^ HIV DUO AG/AB assay on the same analytical VIDAS^®^ platform, was used as the primary comparator to assess improvements in analytical sensitivity and early seroconversion detection.

Samples from the three antigen panels were diluted in normal human plasma that had been delipidated and defibrinated. The limit of detection (LoD) was estimated by linear regression using three p24 antigen dilutions (1, 0.33, and 0.11 IU/mL), each tested in triplicate. The 50 culture supernatants were diluted to approximately 15 pg/mL of p24, as quantified with the VIDAS^®^ HIV p24 II assay (bioMérieux, Marcy L’Etoile, France), resulting in final concentrations falling within a controlled range of 8.1 to 17.2 pg/mL.

A set of 40 seroconversion panels was analyzed to assess early detection performance. Among these, a subset of 17 panels had been previously characterized in a study by Wang et al. [22], enabling additional comparative analysis with the ARCHITECT HIV Ag/Ab Combo and MAGLUMI HIV Ab/Ag Combi assays based on published data.

Testing was performed at the bioMérieux site (Marcy l’Étoile, France) using both VIDAS^®^ HIV DUO AG/AB and VIDAS^®^ HIV DUO Ultra assays, as depicted in Figure 1. For comparative analyses, VIDAS^®^ HIV DUO Ultra index values were normalized by setting the cutoff at 1.00 instead of 0.25.

#### 2.4.2. Sensitivity Evaluation in Established HIV Infection

All samples were tested using the VIDAS^®^ HIV DUO AG/AB assay across two distinct laboratory sites, depending on the type of infection. HIV-1-positive specimens were analyzed at the Croix-Rousse Hospital (Lyon, France), while the majority of HIV-2-positive specimens were tested at the bioMérieux laboratory (Marcy l’Étoile, France); two HIV-2-positive samples were also analyzed at the Croix-Rousse Hospital.

Among the 65 native p24-positive samples included in the diagnostic sensitivity assessment, eight were processed at the Croix-Rousse University Hospital, and the remaining 57 were tested at the bioMérieux site in Marcy l’Étoile.

In addition, the antibody performance panels were entirely tested with VIDAS^®^ HIV DUO AG/AB and VIDAS^®^ HIV DUO Ultra at the bioMérieux site.

#### 2.4.3. Specificity Evaluation

Clinical specificity was assessed using a total of 6063 HIV-negative specimens obtained from four distinct populations, as described in Section 2.2.3. All samples were tested with the VIDAS^®^ HIV DUO AG/AB assay according to the manufacturer’s instructions at the bioMérieux site (Marcy l’Étoile, France). Reactive results were classified as initially reactive (IR) or repeatedly reactive (RR) following duplicate retesting.

For the analysis of antibody and antigen index distributions, HIV-negative blood donor samples from two independent cohorts tested with the VIDAS^®^ HIV DUO AG/AB (*n* = 5407) and VIDAS^®^ HIV DUO Ultra (*n* = 4997) assays were included. Index values were analyzed descriptively using median and interquartile ranges (IQR) to characterize background reactivity and baseline signal distribution in HIV-negative populations. For antigen measurements obtained with the VIDAS^®^ HIV DUO AG/AB assay, ND (Not Determinable) antigen values were assigned a value of 0. These ND results correspond to extremely low antigen signals that do not allow calculation of a reportable antigen index and occur in samples interpreted as HIV-negative according to the assay algorithm. This approach was used solely to enable graphical representation and summary statistics of background reactivity distributions and did not influence clinical sensitivity, specificity, or result interpretation.

The VIDAS^®^ HIV DUO Quick assay, a previous-generation screening test, was included to evaluate the ability of the VIDAS^®^ HIV DUO AG/AB assay to reduce false-positive results observed with earlier VIDAS^®^ assays in routine diagnostic workflows. For this purpose, a subset of 14 HIV-negative samples previously identified as false positives with comparator assays (ARCHITECT HIV Ag/Ab Combo and VIDAS^®^ HIV DUO Quick) was retested using the VIDAS^®^ HIV DUO AG/AB assay at the Rouen University Hospital (Rouen, France), and the proportion of samples reclassified as negative was determined.

Analytical specificity of the VIDAS^®^ HIV DUO AG/AB was evaluated at bioMérieux (Marcy l’Etoile, France) using samples containing antigens or antibodies against various pathogens or corresponding to specific physiologic conditions. All samples were expected to test negative for HIV. Any specimen yielding a reactive result with the VIDAS^®^ HIV DUO AG/AB assay was subsequently tested with the CE-marked VIDAS^®^ HIV DUO Ultra assay to confirm HIV status. Samples found to be HIV-positive with the VIDAS^®^ HIV DUO Ultra assay were further confirmed as positive by Western blot using the New Lav Blot I assay (Bio-Rad, Marnes-la-Coquette, France) and were excluded from the analysis.

#### 2.4.4. Precision Experiments

Precision experiments were conducted at bioMérieux (Marcy l’Etoile, France). Assay precision was evaluated in accordance with the CLSI EP05-A3 guideline [23] using characterized low- and high-positive samples. Within-run precision (repeatability) and within-laboratory precision (within-lot reproducibility) of the VIDAS^®^ HIV DUO AG/AB assay were assessed using samples tested in duplicate, twice daily, over 20 operational testing days on a single VIDAS^®^ instrument calibrated on day 1, resulting in 80 measurements per sample.

### 2.5. Statistical Analysis

Sample size was determined according to the Common Specifications adopted by the European Commission for in vitro diagnostic medical devices [24]. The 95% confidence intervals (95% CI) were computed, either as Wilson Score Confidence Interval if the specificity was in the range [5%, 95%] or as Exact Binomial Confidence Interval otherwise, using the SAS Enterprise Guide 8.2 software. For the comparison of first-reactive days between VIDAS^®^ HIV DUO AG/AB and VIDAS^®^ HIV DUO Ultra across seroconversion panels, paired differences were analyzed using a one-sided Wilcoxon signed-rank test. Only non-zero paired differences were included in the analysis. A significance level of 0.05 was used. Assay precision was evaluated according to the Clinical and Laboratory Standards Institute (CLSI) EP05-A3 guideline using variance component analysis and restricted maximum likelihood (REML), implemented in SAS Enterprise Guide 8.2 software.

## 3. Results

### 3.1. Sensitivity in Early HIV Infection

Analytical sensitivity and early seroconversion performance were first assessed using WHO standards, culture supernatants, and seroconversion panels.

VIDAS^®^ HIV DUO AG/AB showed a lower estimated p24 antigen LoD than VIDAS^®^ HIV DUO Ultra in this study on both WHO standards (NIBSC 22/230 and 90/636), with an LoD approximately two-fold lower than that of the VIDAS^®^ HIV DUO Ultra (0.3 IU/mL versus 0.5–0.6 IU/mL, respectively).

**Figure 2:**
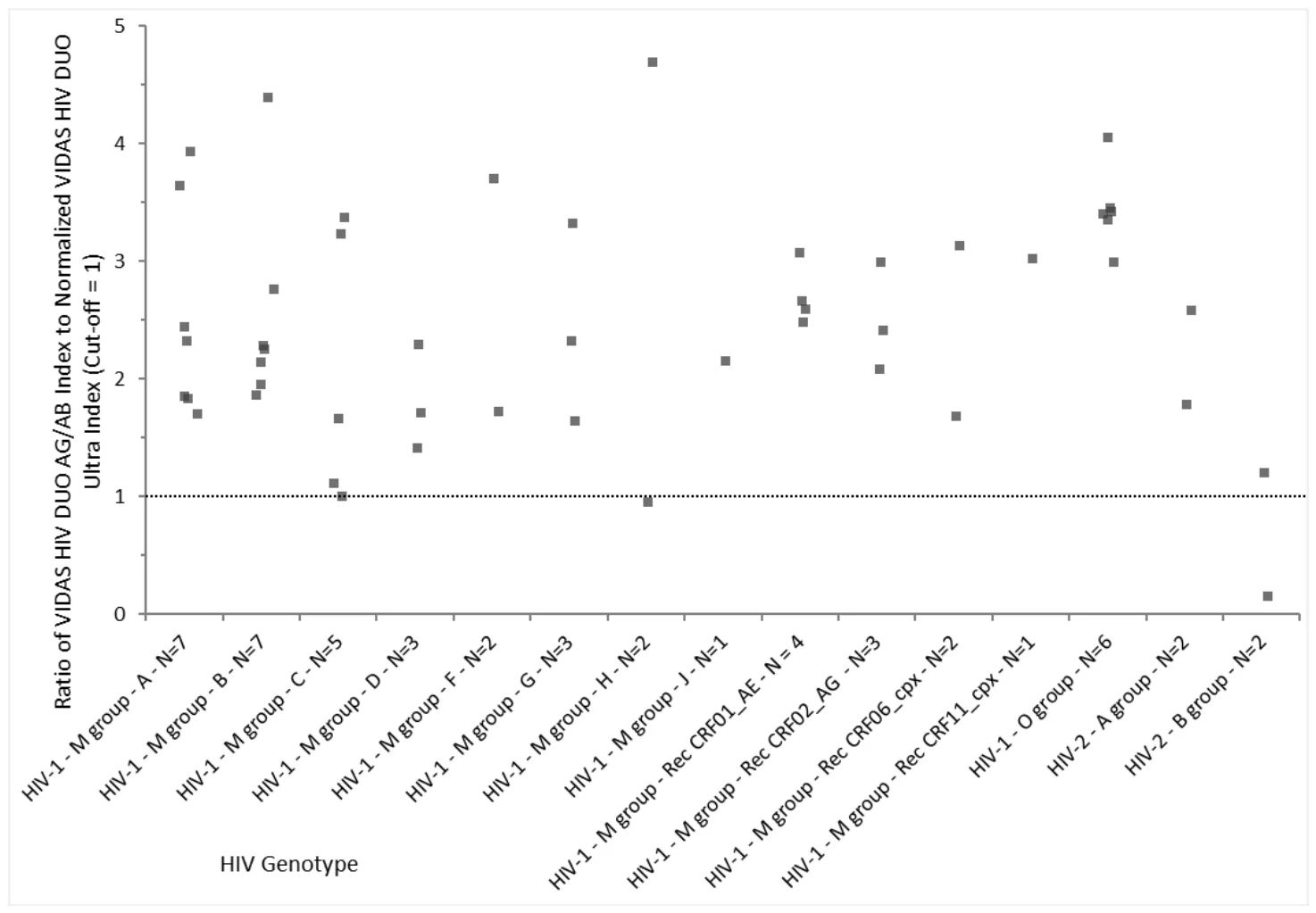
Comparison of HIV-1 p24 antigen reactivity between the VIDAS^®^ HIV DUO AG/AB and VIDAS^®^ HIV DUO Ultra assays across multiple genotypes (cell culture supernatants). Individual data points represent the ratio of the VIDAS^®^ HIV DUO AG/AB p24 index to the normalized VIDAS^®^ HIV DUO Ultra p24 index for each HIV culture supernatant tested. For comparative analyses, VIDAS^®^ HIV DUO Ultra index values were normalized by setting the cut-off at 1.00 instead of 0.25. The dotted horizontal line indicates a ratio of 1.00, corresponding to equivalent reactivity between the two assays. Ratios above 1.00 reflect higher relative p24 reactivity with the VIDAS^®^ HIV DUO AG/AB assay.

The comparison of HIV-1 p24 antigen reactivity between the VIDAS^®^ HIV DUO AG/AB and VIDAS^®^ HIV DUO Ultra assays across multiple HIV genotypes is shown in Figure 2. For each genotype category, individual points represent the ratio of the VIDAS^®^ HIV DUO AG/AB index to the normalized VIDAS^®^ HIV DUO Ultra index. Ratio values ranged from approximately 1 to above 4 for 48/50 (96.0%) samples across all genotypes, with each category displaying a dispersed distribution of results. Under the experimental conditions used in this study, these results indicate higher relative p24 antigen reactivity for most tested samples with the VIDAS^®^ HIV DUO AG/AB assay compared with VIDAS^®^ HIV DUO Ultra across a broad range of HIV genotypes.

Early seroconversion performance was assessed using 40 seroconversion panels tested with both the VIDAS^®^ HIV DUO AG/AB and the VIDAS^®^ HIV DUO Ultra assays. As shown in Table 1, the VIDAS^®^ HIV DUO AG/AB assay detected HIV infection one bleed earlier in 11 panels (27.5%), two bleeds earlier in 1 panel (2.5%), and three bleeds earlier in 1 panel (2.5%) compared with the VIDAS^®^ HIV DUO Ultra assay. Moreover, VIDAS^®^ HIV DUO AG/AB detected 17 additional bleeds compared with VIDAS^®^ HIV DUO Ultra across the 358 bleeds included in the 40 seroconversion panels. Detailed panel-by-panel bleed-level results are provided in Table S1.

A comparison with the ARCHITECT HIV Ag/Ab Combo (Abbott Diagnostics, Abbott Park, IL, USA) and the MAGLUMI HIV Ab/Ag Combi (Snibe Diagnostics, Shenzhen, China) assays, based on a subset of 17 seroconversion panels previously characterized by Wang et al. [22], showed that VIDAS^®^ HIV DUO AG/AB detected infection one bleed earlier in 8 panels (47.1%) compared with ARCHITECT. Similarly, VIDAS^®^ HIV DUO AG/AB detected HIV infection one bleed earlier in 7 panels (41.2%) compared with MAGLUMI (Table 1). All other panels were detected at the same bleed between the two assays, except for one panel (HIV9031), which was detected one bleed later with VIDAS^®^ HIV DUO AG/AB; in this case, the antigen signal was close to the cut-off (S/CO = 0.94).

Analysis of the first-reactive day for each seroconversion panel showed that the VIDAS^®^ HIV DUO AG/AB assay demonstrated either equivalent or earlier detection of HIV infection compared with VIDAS^®^ HIV DUO Ultra (Table 2). Among the 40 seroconversion panels, 27 (67.5%) showed identical first-reactive days, whereas 13 (32.5%) were detected earlier with VIDAS^®^ HIV DUO AG/AB. No panel was detected later with VIDAS^®^ HIV DUO AG/AB. Analysis of the 13 informative paired differences demonstrated a statistically significant shift toward earlier detection with VIDAS^®^ HIV DUO AG/AB (one-sided Wilcoxon signed-rank test, *n* = 13 non-zero pairs, *p* < 0.001). Twenty-seven panel pairs showed no difference in first-reactive day and therefore did not contribute to the Wilcoxon signed-rank statistics. The cumulative gain in first-reactive-day detection by VIDAS^®^ HIV DUO AG/AB across all panels was 60 days.

For the subset of 17 seroconversion panels previously characterized by Wang et al. [22], VIDAS^®^ HIV DUO AG/AB also demonstrated a tendency toward earlier detection than the ARCHITECT HIV Ag/Ab Combo and MAGLUMI HIV Ab/Ag Combi assays. Earlier detection was observed in 8 (47.1%) and 7 (41.2%) panels, respectively, corresponding to cumulative gains of 29 and 19 days (Table 3). Representative examples of these earlier detection patterns are provided in Table S3, which presents detailed antigen and antibody reactivity profiles for selected seroconversion panels spanning different stages of early HIV infection.

Further insight into assay reactivity patterns during early infection is provided in Tables S2 and S3, which present illustrative antibody and antigen index profiles for selected seroconversion panel members tested with VIDAS^®^ HIV DUO AG/AB in comparison with VIDAS^®^ HIV DUO Ultra and, where available, the ARCHITECT HIV Ag/Ab Combo assay, with Fiebig stage classifications provided for selected panels in Table S3. These Supplementary Data demonstrate that VIDAS^®^ HIV DUO AG/AB detected a higher number of reactive samples and identified reactivity at earlier time points for several panel members, including cases with low-level antibody or antigen signals that remained non-reactive or non-determinable with comparator assays.

### 3.2. Sensitivity in Established HIV Infection

A total of 634 HIV positive specimens, including 436 anti-HIV-1-positive, 133 anti-HIV-2-positive and 65 HIV-1 p24 antigen-positive samples, were tested with the VIDAS^®^ HIV DUO AG/AB assay. All 634 samples (100%) were correctly detected as positive, as summarized in Table 4.

Within the anti-HIV-1 panel, all 436 specimens were reactive, spanning a wide diversity of HIV-1 genotypes (Table 4). Similarly, all 133 HIV-2 antibody-positive specimens were detected by the assay, including 48 individuals receiving antiretroviral treatment (Table 4).

In addition, all 65 native HIV-1 p24-positive specimens—originating from various clinical and commercial sources—were detected as reactive, confirming complete detection within this antigen-positive subset (Table 4).

Across the antibody and antigen performance panels, VIDAS^®^ HIV DUO AG/AB identified 27 reactive samples, whereas VIDAS^®^ HIV DUO Ultra detected 24 reactive samples. The additional detections primarily corresponded to samples with low reactivity, characterized by S/CO values close to the assay threshold (i.e., near 1.00) and including early infection specimens with borderline antigen or antibody responses (Table S4).

### 3.3. Specificity

Specificity of the VIDAS^®^ HIV DUO AG/AB assay was assessed using a total of 6063 HIV-negative specimens collected from four distinct populations (Table 5). Among 5407 blood donors, two initially reactive and one repeatedly reactive samples were observed, yielding a specificity of 99.98% (95% CI: 99.90–100.00%). Among 251 hospitalized patients, one repeatedly reactive sample was reported, corresponding to a specificity of 99.60% (95% CI: 97.80–99.99%). Within the 282 high-risk-behavior subjects, including 75 individuals receiving pre-exposure prophylaxis (PrEP; Truvada), one repeatedly reactive result was observed, giving a specificity of 99.65% (95% CI: 98.04–99.99%). All 123 samples from pregnant women tested negative, resulting in a specificity of 100.00% (95% CI: 97.00–100.00%).

Across all 6063 negative samples, the assay achieved an overall specificity of 99.95% (95% CI: 99.90–99.99%), with only 4 initially reactive samples and 3 repeatedly reactive samples (Table 5).

**Figure 3:**
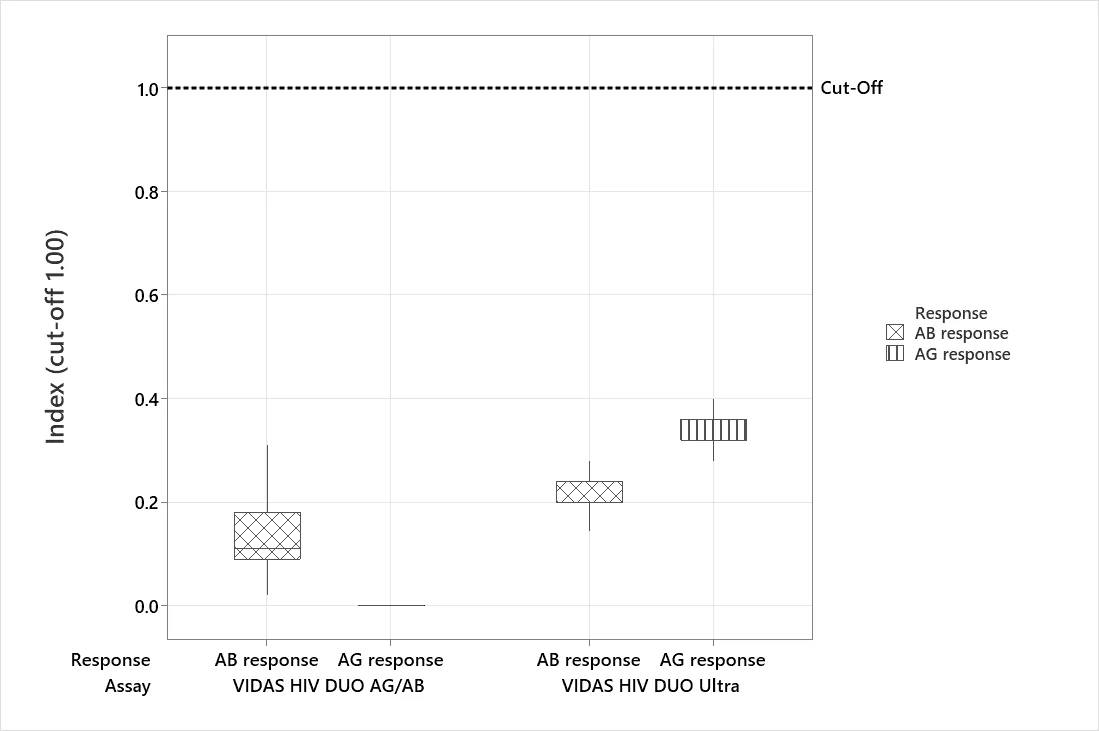
Distribution of antibody and antigen response indexes in HIV-negative samples from two independent blood-donor cohorts tested with the VIDAS^®^ HIV DUO AG/AB and VIDAS^®^ HIV DUO Ultra assays. AB and AG response index distributions were obtained from HIV-negative blood-donor samples tested negative with the VIDAS^®^ HIV DUO AG/AB assay (*n* = 5406) and the VIDAS^®^ HIV DUO Ultra assay (*n* = 4997). The two assays were evaluated using independent blood-donor cohorts collected from different French Blood Establishment (EFS) regions and at different time periods. All samples were confirmed HIV-negative according to the local screening algorithms, including fourth-generation serological testing and viral genome detection. Index values represent S/CO measurements for AB and AG responses. For antigen measurements obtained with the VIDAS^®^ HIV DUO AG/AB assay, not determinable (ND) values were assigned a value of 0 for descriptive analysis of background reactivity distributions. The distributions illustrate baseline signal behavior and assay background reactivity in HIV-negative populations.

The distribution of antibody and antigen response indexes in HIV-negative samples from two independent blood-donor cohorts tested with the VIDAS^®^ HIV DUO AG/AB and VIDAS^®^ HIV DUO Ultra assays is shown in Figure 3. Median index values were lower for the VIDAS^®^ HIV DUO AG/AB assay than for the VIDAS^®^ HIV DUO Ultra assay. The median (IQR) antibody and antigen index values were 0.11 (0.09–0.18) and 0.00 (0.00–0.00) for VIDAS^®^ HIV DUO AG/AB, and 0.20 (0.20–0.24) and 0.32 (0.32–0.36) for VIDAS^®^ HIV DUO Ultra, respectively (Figure 3).

To further assess the ability of the VIDAS^®^ HIV DUO AG/AB assay to minimize non-specific reactivity, false-positive samples previously identified with comparator assays (*n* = 14), as described in the non-specific reactivity assessment, were retested using the VIDAS^®^ HIV DUO AG/AB assay. As shown in Table 6, among the 10 false-positive samples initially identified with the ARCHITECT HIV AG/AB Combo assay, 8 were correctly classified as negative by the VIDAS^®^ HIV DUO AG/AB assay (80% corrective performance). Similarly, among the 4 false-positive samples identified by the VIDAS^®^ HIV DUO Quick assay, 3 were reclassified as negative (75% corrective performance).

The analytical specificity of the VIDAS^®^ HIV DUO AG/AB assay was evaluated using 272 samples containing antibodies, antigens, or physiological conditions known to potentially interfere with HIV immunoassays (Table 7). The panel included specimens positive for a range of viral infections (e.g., HAV, HBV, HCV, HEV, HSV, HTLV, CMV, EBV, influenza, rubella, SARS-CoV-2), parasitic or bacterial infections (toxoplasmosis, syphilis), autoimmune markers (ANA, RF), human anti-mouse antibodies, and samples from vaccinated individuals (hepatitis B, influenza, SARS-CoV-2).

Across all 19 categories, no cross-reactive results were observed (0/272), yielding an analytical specificity of 100% with a 95% confidence interval of 98.65–100.00%.

### 3.4. Precision

Repeatability and within-lot reproducibility were assessed using low- and high-level samples for both antibody and antigen responses (Table 8). Across the four conditions tested, mean index values ranged from 2.71 to 11.71, with repeatability coefficients of variation (CVs) between 2.4% and 2.7% and within-lot reproducibility CVs between 2.9% and 3.4%. Thus, all precision estimates remained below 3.5% CV.

## 4. Discussion

This study evaluated the analytical and clinical performance of VIDAS^®^ HIV DUO AG/AB, a new fourth-generation test designed to enhance early HIV diagnosis. The results demonstrate that VIDAS^®^ HIV DUO AG/AB provides improved analytical sensitivity, earlier seroconversion detection, higher specificity, broader inclusivity, and greater precision than its predecessor, VIDAS^®^ HIV DUO Ultra. Comparisons with previously published seroconversion panel data further suggest favorable early-detection performance relative to selected fourth-generation assays.

The use of multiple comparator assays reflects complementary evaluation strategies. In particular, the VIDAS^®^ HIV DUO Ultra assay served as the primary comparator to assess performance improvements over the established VIDAS^®^ platform, while comparisons with ARCHITECT HIV Ag/Ab Combo and MAGLUMI HIV Ag/Ab Combi provided a broader perspective on performance relative to widely used fourth-generation assays. In addition, the inclusion of the VIDAS^®^ HIV DUO Quick assay allowed evaluation of the reduction in false-positive results observed with previous-generation assays in routine practice.

The superior analytical sensitivity of the VIDAS^®^ HIV DUO AG/AB assay was clearly demonstrated through its lower limit of detection for p24 antigen estimated at 0.3 IU/mL using both NIBSC international HIV-1 p24 subtype B standards. Therefore, the VIDAS^®^ HIV DUO AG/AB assay is among the most sensitive assays currently reported in the literature, with Access HIV combo V2 (0.39 IU/mL), Elecsys HIV Duo (0.35 IU/mL), or BioPlex 2200 HIV Ag-Ab (0.21 IU/mL), outperforming ARCHITECT HIV Combo (0.72 IU/mL) and MAGLUMI HIV Combi (0.78 IU/mL) assays [19,21,22]. This result is further supported by the analysis of 50 HIV culture supernatants, for which the assay showed higher relative p24 reactivity than the VIDAS^®^ HIV DUO Ultra assay across HIV-1 group M subtypes and circulating recombinant forms, confirming enhanced detection over a broad range of viral lineages. Although one HIV-2 culture supernatant showed reduced reactivity, the signal remained detectable. Given the rarity of HIV-2 primary infections and the limited availability of well-characterized seroconversion samples, the likelihood of missing such early infections is expected to be low. Moreover, as HIV-2 antigen detection is not required by current Common Specifications, this isolated observation does not affect the overall performance trend.

The improved analytical performance translated into earlier detection during seroconversion. The VIDAS^®^ HIV DUO AG/AB assay identified infection at earlier bleeds in more than 30% of the evaluated panels compared with the VIDAS^®^ HIV DUO Ultra, the ARCHITECT HIV Ag/Ab Combo and the MAGLUMI HIV Ag/Ab Combi assays. It should be noted that a single seroconversion panel (HIV9031) showed detection one bleed later with the VIDAS^®^ HIV DUO AG/AB assay compared with the MAGLUMI assay. In this case, the antigen index value obtained with VIDAS^®^ HIV DUO AG/AB was close to the cut-off (S/CO = 0.94), suggesting that the difference may be attributable to signal variability around the assay threshold. Notably, although most seroconversion panels showed identical first-reactive days with VIDAS^®^ HIV DUO AG/AB and VIDAS^®^ HIV DUO Ultra, approximately one-third of the panels were detected earlier with VIDAS^®^ HIV DUO AG/AB, while none were detected later. This distribution was supported by a statistically significant shift toward earlier detection with VIDAS^®^ HIV DUO AG/AB in paired first-reactive-day comparisons (Wilcoxon signed-rank test, *p* < 0.001). This corresponded to the detection of 17 additional bleeds and a cumulative gain of 60 days across the 40 evaluated panels. These findings suggest that the benefit of VIDAS^®^ HIV DUO AG/AB is concentrated in a subset of panels representing the earliest phases of infection, where even modest gains in detection timing may have important clinical implications, as early diagnosis is critical for timely clinical management and transmission prevention [9,25,26].

In line with these findings, a recent study by Mchantaf et al. conducted in a cohort of 77 well-characterized HIV patients diagnosed during primary infection and stratified according to Fiebig stages demonstrated the robust performance of the VIDAS^®^ HIV DUO AG/AB assay for early HIV diagnosis, with all evaluated samples correctly identified as reactive, including those from individuals at Fiebig stage II [27]. However, the cohort predominantly consisted of patients in later acute or early seroconversion phases (Fiebig stages IV to VI) with a high proportion of Not Determinable (ND) antigen or antibody responses, which, according to the authors, may impact the classification of Fiebig stages. Importantly, ND responses should not be interpreted as indeterminate or invalid results. Rather, they reflect the assay design, whereby very high concentrations of either HIV-1 p24 antigen or anti-HIV antibodies may prevent accurate calculation of the complementary response, while a valid final interpretation remains possible based on the unmasked marker. Our Supplementary Data. (Tables S2–S4) illustrate how a strong signal from one response may mask the reactivity of the other without compromising early diagnosis. Specifically, Table S3 presents results from three seroconversion panels (PRB969, PRB971, and PRB974) characterized according to Fiebig stages. Notably, the VIDAS^®^ HIV DUO AG/AB assay detected reactive antigen index values as early as Fiebig stage I across all panels, highlighting its high sensitivity for detecting early p24 antigenemia. Our results further suggest that highly sensitive antigen detection may enable infection diagnosis within the earliest phases defined by conventional staging approaches, demonstrating the ability of the assay to detect infection at very early time points. In addition, antigen positivity persisted up to Fiebig stage III, while the antibody response remained ND. These observations demonstrate the ability of the assay to maintain antigen detection during the transition toward seroconversion. These findings are consistent with the vendor data, which indicate high p24 positivity at these stages, along with minimal antibody-only reactivity at Fiebig stage III. Samples from Fiebig stages IV and V frequently showed reactive antibody index values associated with masked ND antigen responses, reflecting the progressive replacement of antigenemia by a dominant antibody response during seroconversion. Similarly, data presented in Table S4 showed that most samples from late Fiebig stage V within the AccuSet HIV-1 early infection panel (0800-0394) displayed reactive antibody index values, while antigen responses were ND, consistent with progression through seroconversion. Given the high p24 analytical sensitivity of VIDAS^®^ HIV DUO AG/AB assay, reactive antigen responses may persist slightly longer than with the previous VIDAS^®^ HIV DUO Ultra assay. This extended antigen detection window may represent an advantage in screening settings, as the presence of a reportable antigen response facilitates the identification of acute HIV infection.

Altogether, the VIDAS^®^ HIV DUO AG/AB assay demonstrated improved analytical sensitivity and earlier seroconversion detection compared with VIDAS^®^ HIV DUO Ultra. Furthermore, indirect comparisons based on published studies suggested a tendency toward earlier detection with VIDAS^®^ HIV DUO AG/AB than with the ARCHITECT HIV Ag/Ab Combo and MAGLUMI HIV Ab/Ag Combi assays in selected seroconversion panels. When contextualized with previously published data [19,21,22], these findings suggest that the VIDAS^®^ HIV DUO AG/AB assay performs at least comparably to other high-performance fourth-generation assays, with a tendency toward earlier detection in seroconversion panels.

Inclusivity testing confirmed that the assay reliably detected all 634 HIV-positive specimens evaluated in this study, including 436 HIV-1-antibody positive, 133 HIV-2 antibody-positive, and 65 HIV-1 p24 antigen-positive samples. These specimens encompassed a broad spectrum of viral diversity, covering HIV-1 group M subtypes, circulating recombinant forms, HIV-1 group O, HIV-2 groups A and B, as well as samples of unknown genotype. The complete detection of both antibody- and antigen-positive samples supports the breadth of analytical recognition of the assay and underscores its suitability for the diagnosis of infections involving genetically diverse HIV strains [28,29,30].

The overall specificity (99.95%; 95% CI: 99.90–99.99%) obtained across all four tested populations confirms the reliability of the VIDAS^®^ HIV DUO AG/AB assay to accurately identify HIV-negative samples in a broad range of clinical settings. The near-perfect specificity observed in blood donors aligns with expectations for a population routinely used to benchmark screening assays, while the strong performance in hospitalized patients and high-risk individuals demonstrates robustness even in groups where cross-reactivity or non-specific immune stimulation can be more common. Importantly, the absence of false-positive results among pregnant women suggests that the assay performs reliably in this physiologically distinct population, where serological assays can sometimes show reduced specificity [31].

The comparison of index distributions for antibody and antigen responses in HIV-negative samples further emphasizes the analytical advantages of the VIDAS^®^ HIV DUO AG/AB assay. In this evaluation, the VIDAS^®^ HIV DUO AG/AB showed consistently lower median index values for both responses compared with the VIDAS^®^ HIV DUO Ultra assay, indicating reduced background signal and a more controlled assay baseline. Lower index distributions provide a wider specificity margin relative to the cut-off, supporting a more reliable distinction between negative samples and those with very low reactivity. It should be noted that ND antigen results observed in HIV-negative samples corresponded to extremely low signals and were assigned a value of 0 solely for descriptive analysis of background reactivity distributions. This data-handling approach did not affect clinical performance estimates or result interpretation.

In line with this, the absence of cross-reactivity across a broad range of infectious, immunological and post-vaccination conditions further demonstrates the strong analytical specificity of the VIDAS^®^ HIV DUO AG/AB assay. Importantly, the evaluation included multiple conditions associated with acute infections characterized by IgM responses, none of which produced a reactive result. Similar findings have been reported for other high-performance fourth-generation assays exposed to large cross-reactivity challenge panels [32]. The evaluation covered clinically relevant sources of potential interference—including acute viral infections, autoimmune markers, human anti-animal antibodies, and vaccine-induced immune responses—without any observed false reactivity. Moreover, the VIDAS^®^ HIV DUO AG/AB assay effectively corrected most of the false-positive results generated by comparator assays, reclassifying 8 out of 10 samples (80%) initially reactive with the ARCHITECT HIV Ag/Ab Combo assay and 3 out of 4 samples (75%) identified with the VIDAS^®^ HIV DUO Quick assay as negative. These data further highlight the robustness of the VIDAS^®^ HIV DUO AG/AB assay in minimizing non-specific reactivity and support its value as a reliable tool in diagnostic workflows where it is essential to avoid false-positive results.

Overall, these findings indicate that the VIDAS^®^ HIV DUO AG/AB assay maintains excellent specificity across heterogeneous populations, supporting its suitability for both large-scale screening and clinical diagnostic applications and highlighting its potential value as a secondary confirmatory tool in settings requiring enhanced result confidence.

Precision studies demonstrated low variability, with repeatability and within-lot reproducibility coefficients of variation below 3.5% across antibody and antigen responses at both low and high index levels. These results confirm that the assay delivers consistent and stable performance across its analytical range.

A key strength of this study lies in the comprehensive and robust evaluation of the VIDAS^®^ HIV DUO AG/AB assay across a large and diverse set of samples representative of real-world clinical practice. The assay was assessed using multiple clinically and epidemiologically relevant populations, including blood donors, hospitalized patients, high-risk individuals, and pregnant women, as well as extensive analytical materials such as international reference standards, HIV culture supernatants covering a wide range of groups, subtypes and circulating recombinant forms, and native antigen- and antibody-positive clinical specimens. This broad analytical and clinical scope supports the generalizability of the results and provides strong evidence for assay sensitivity, inclusivity, specificity, and precision across genetically diverse HIV strains and heterogeneous testing settings.

Several limitations of this study should be acknowledged. First, analytical sensitivity was not evaluated using the WHO 16/210 reference panel, which enables standardized group- and subtype-specific characterization; however, a dedicated evaluation using this panel is currently under preparation. Second, early infection performance was primarily assessed using subtype B seroconversion panels, reflecting the limited availability of subtype-diverse native seroconversion samples, which may restrict extrapolation of these findings to all global HIV subtypes. Third, a substantial proportion of the specimens included in the study were archived samples collected retrospectively. Although these samples enabled the evaluation of a broad range of HIV genotypes and clinical situations, their retrospective nature may not fully reflect current testing conditions. Fourth, comparator performance for the ARCHITECT HIV Ag/Ab Combo and MAGLUMI HIV Combi assays was derived from published data rather than from parallel retesting under identical experimental conditions. Fifth, background index distribution analyses for the VIDAS^®^ HIV DUO AG/AB and VIDAS^®^ HIV DUO Ultra assays were conducted using different blood donor cohorts. Although comparison within a single cohort would have been preferable, quantitative Elecsys HIV Duo index values generated during routine donor qualification were not available for retrospective analysis. Consequently, the comparison was restricted to datasets available from the VIDAS^®^ HIV DUO AG/AB and VIDAS^®^ HIV DUO Ultra evaluations. All blood donors included in the study were confirmed HIV-negative using serological and NAT screening, supporting the interpretation that the observed differences mainly reflect assay-specific background signal characteristics. Furthermore, the large number of blood-donor samples included in both cohorts contributes to the robustness of the observed index distribution patterns. Finally, the study population was recruited predominantly from French clinical cohorts. Despite this caveat, the evaluation specimens encompassed a broad diversity of HIV-1 and HIV-2 variants, including multiple group M subtypes, circulating recombinant forms, group O viruses, and HIV-2 samples. Nevertheless, additional evaluations performed in geographically distinct populations and independent clinical settings are warranted to further confirm the generalizability of these findings. Despite these limitations, the consistency of results across analytical and clinical endpoints supports the robustness of the overall conclusions and the relevance of the assay for routine clinical use.

## 5. Conclusions

Overall, the findings of this study support the use of the VIDAS^®^ HIV DUO AG/AB assay as a sensitive and reliable fourth-generation HIV screening assay, with improved analytical and clinical performance compared with its predecessor, VIDAS^®^ HIV DUO Ultra. In addition, indirect comparisons based on previously published seroconversion panel data further suggest favorable early-detection performance relative to the ARCHITECT HIV Ag/Ab Combo and MAGLUMI HIV Ab/Ag Combi assays. The assay demonstrated robust performance across a broad range of HIV-1 and HIV-2 variants and showed particular value for the detection of early HIV infection. Additional evaluations performed in independent and geographically diverse populations would further strengthen evidence of its performance across different epidemiological contexts.

## Figures and Tables

**Table 1:** Performance of VIDAS^®^ HIV DUO AG/AB versus comparator assays in early seroconversion detection.

Comparator Assay (Versus VIDAS^®^ HIV DUO AG/AB)	Number of Panels Tested	Panels Detected Earlier by VIDAS^®^ HIV DUO AG/AB		
		1 Bleed Earlier *n* (%)	2 Bleeds Earlier *n* (%)	3 Bleeds Earlier *n* (%)
VIDAS^®^ HIV DUO Ultra	40	11 (27.5)	1 (2.5)	1 (2.5)
ARCHITECT HIV Ag/Ab Combo *	17	8 (47.1)	0 (0.0)	0 (0.0)
MAGLUMI HIV Ag/Ab Combi *	17 **	7 (41.2)	0 (0.0)	0 (0.0)

Values indicate the number and percentage of seroconversion panels for which VIDAS^®^ HIV DUO AG/AB showed earlier detection than the named comparator assay. * From Wang et al., 2024 [22]. ** One seroconversion panel (HIV9031) showed a one-bleed later detection with VIDAS^®^ HIV DUO AG/AB compared with the MAGLUMI assay. In this case, the antigen index value obtained with VIDAS^®^ HIV DUO AG/AB was close to the cut-off (S/CO = 0.94).

**Table 2:** Comparison of the 1st reactive day (days from the 1st bleed) obtained with the VIDAS^®^ HIV DUO AG/AB and VIDAS^®^ HIV DUO Ultra assays across 40 HIV seroconversion panels.

	1st Reactive Day		
Panel ID	VIDAS^®^ HIV DUO AG/AB (Days)	VIDAS^®^ HIV DUO Ultra (Days)	Difference Between VIDAS^®^ HIV DUO AG/AB and Ultra (Days)
PRB925	44	44	0
PRB926	7	7	0
PRB935	28	28	0
PRB941	9	9	0
PRB943	7	7	0
PRB944	2	7	−5
PRB945	7	7	0
PRB947	9	9	0
PRB951	8	8	0
PRB952	10	10	0
PRB953	3	3	0
PRB954	14	17	−3
PRB955	3	3	0
PRB958	7	7	0
PRB967	7	17	−10
PRB969	61	70	−9
PRB971	2	7	−5
PRB973	2	7	−5
PRB974	7	9	−2
0600-0270	27	30	−3
0600-0271	3	7	−4
0600-0272	18	18	0
HIV6240	21	23	−2
HIV6246	49	52	−3
HIV9011	38	38	0
HIV9012	16	16	0
HIV9013	23	23	0
HIV9015	30	30	0
HIV9017	0 (AG) * 17 (AB)	- 24 (AB)	−7 *
HIV9018	25	25	0
HIV9021	47	47	0
HIV9022	23	23	0
HIV9025	85	85	0
HIV9031	146	146	0
HIV9032	22	24	−2
HIV9034	46	46	0
HIV9076	66	66	0
HIV9077	45	45	0
HIV9079	40	40	0
HIV12008	23	23	0
Total delayed days on 40 panels	1047	1107	−60

All reported values correspond to the number of days from the 1st bleed within each seroconversion panel. Negative values indicate earlier detection by VIDAS^®^ HIV DUO AG/AB compared with VIDAS^®^ HIV DUO Ultra. * 9017−01 (D0) is positive AG, then 9017−02/03/04/05 (D3–D14) back to negative AG/AB, and 9017−06 (D17) and next AB-positive. The difference was calculated using AB difference detection.

**Table 3:** Comparison of 1st reactive day (days from the 1st bleed) obtained with the VIDAS^®^ HIV DUO AG/AB, ARCHITECT HIV Ag/Ab Combo, and MAGLUMI HIV Ab/Ag Combi assays across 17 HIV seroconversion panels previously reported by Wang et al. [22].

	1st Reactive Day				
	VIDAS^®^ HIV DUO AG/AB (Days)	ARCHITECT HIV Ag/Ab Combo * (Days)	MAGLUMI HIV Ab/Ag Combi * (Days)	Difference Between	
				VIDAS^®^ & ARCHITECT * (Days)	VIDAS^®^ & MAGLUMI * (Days)
PRB945	7	13	13	−6	−6
PRB955	3	3	3	0	0
PRB969	61	63	63	−2	−2
PRB973	2	7	7	−5	−5
0600-0271	3	7	7	−4	−4
HIV9011	38	38	38	0	0
HIV9012	16	16	16	0	0
HIV9013	23	25	23	−2	0
HIV9018	25	28	28	−3	−3
HIV9021	47	47	47	0	0
HIV9022	23	25	25	−2	−2
HIV9031	146	146	138	0	+8 **
HIV9034	46	46	46	0	0
HIV9076	66	66	66	0	0
HIV9077	45	45	45	0	0
HIV9079	40	40	40	0	0
HIV12008	23	28	28	−5	−5
Total delayed days on 17 panels	614	643	633	−29	−19

All reported values correspond to the number of days from the 1st bleed within each seroconversion panel. Negative values indicate earlier detection by VIDAS^®^ HIV DUO AG/AB compared with the corresponding comparator assay. * Data from Wang et al. [22]. ** The value +8 corresponds to panel HIV9031, which is the only panel showing a one-bleed later detection with VIDAS^®^ HIV DUO AG/AB compared with the MAGLUMI assay. The antigen index value for this sample was borderline (S/CO = 0.94).

**Table 4:** Comprehensive detection of diverse HIV-1 and HIV-2 genotypes by VIDAS^®^ HIV DUO AG/AB.

	Genotype	Samples	Positive	Negative
HIV-1 Ab positive	-	436	436	0
	A	7	7	0
	A1	15	15	0
	A2	1	1	0
	B	159	159	0
	C	23	23	0
	D	6	6	0
	CRF01_AE	9	9	0
	CRF02_AG	123	123	0
	CRF06_cpx	9	9	0
	CRF11_cpx	10	10	0
	CRF12_BF	2	2	0
	CRF13_cpx	3	3	0
	CRF14_BG	3	3	0
	CRF18_cpx	1	1	0
	CRF19_cpx	1	1	0
	CRF22_01A1	2	2	0
	CRF36_cpx	1	1	0
	CRF44_BF	1	1	0
	CRF45_cpx	1	1	0
	F	4	4	0
	F1	6	6	0
	F2	12	12	0
	G	11	11	0
	J	1	1	0
	K	1	1	0
	Group O	11	11	0
	Unknown	13	13	0
HIV-1 Ag-positive HIV-2 Ab-positive	Unknown -	65 133	65 133	0 0
Total		634	634	0

**Table 5:** Specificity of the VIDAS^®^ HIV DUO AG/AB assay across diverse patient populations.

Populations	VIDAS^®^ HIV DUO AG/AB			
	Samples	IR/RR	Specificity	95% CIs
Blood donors	5407	2/1	99.98%	99.90–100.00%
Hospitalized patients	251	1/1	99.60%	97.80–99.99%
High-risk behavior subjects	282	1/1	99.65%	98.04–99.99%
Pregnant women	123	0/0	100.00%	97.00–100.00%
Total	6063	4/3	99.95%	99.90–99.99%

Abbreviations: IR, initially reactive; RR, repeatedly reactive.

**Table 6:** Comparative summary of false-positive results across assays.

Assay	Number of False Positives Detected	Number Correctly Classified as Negative by VIDAS^®^ HIV DUO AG/AB	Corrective Performance
ARCHITECT HIV Ag/Ab Combo	10	8	80%
VIDAS^®^ HIV DUO Quick	4	3	75%

**Table 7:** Analytical specificity of VIDAS^®^ HIV DUO AG/AB in samples with potentially cross-reacting antibodies and/or antigens.

Potentially Interfering Infections or Conditions	Proportion of Cross-Reactive Samples
Hepatitis A Virus (HAV)	0/13
Hepatitis B Virus (HBV)	0/47
Hepatitis C Virus (HCV)	0/24
Hepatitis E Virus (HEV)	0/12
Herpes Simplex Virus (HSV)	0/12
Human T-cell Lymphotropic Virus (HTLV)	0/10
Cytomegalovirus (CMV)	0/10
Epstein-Barr Virus (EBV)	0/20
Influenza A Virus	0/11
Rubella Virus	0/13
SARS-CoV-2 Virus	0/10
Toxoplasmosis	0/9
Syphilis	0/10
Hepatitis B vaccinated	0/9
Influenza A/B vaccinated	0/10
SARS-CoV-2 vaccinated	0/10
Anti-Nuclear Antibodies (ANA)	0/20
Rheumatoid Factor (RF)	0/12
Human Anti-Mouse Antibodies (HAMA)	0/10
TOTAL	0/272
Analytical specificity	100.00%
95% CI	98.65–100.00%

**Table 8:** Repeatability and within-lot reproducibility of the VIDAS^®^ HIV DUO AG/AB assay.

Response	Level	***N***	Mean Index Value	Repeatability (Within-Run Precision)		Within-Lot Reproducibility (Within-Laboratory Precision)	
				SD	CV (%)	SD	CV (%)
AB	Low	80	2.71	0.07	2.4	0.09	3.3
	High	80	10.31	0.27	2.6	0.35	3.4
AG	Low	80	2.91	0.08	2.7	0.10	3.3
	High	80	11.71	0.31	2.7	0.34	2.9

Abbreviations: SD, Standard Deviation, CV, Coefficient of Variation.

## Data Availability

The original contributions presented in this study are included in the article/Supplementary Material. Further inquiries can be directed to the corresponding author.

## Supplementary Materials

The following supporting information can be downloaded at: https://www.mdpi.com/article/10.3390/diagnostics16182954/s1, Figure S1: Algorithm for HIV infection screening at the Croix-Rousse Hospital (Lyon, France); Figure S2: Algorithm for HIV infection screening at the QBD (Qualification Biologique du Don) sites of the French Blood Establishments; Table S1: Comparison of the number of bleeds detected with the VIDAS^®^ HIV DUO AG/AB and VIDAS^®^ HIV DUO Ultra assays across 40 HIV seroconversion panels used for early infection sensitivity evaluation. AG/Ag antigen, AB/Ab antibodies; Table S2: Representative examples of antibody and antigen reactivity profiles from selected HIV seroconversion panels illustrating earlier or enhanced detection by VIDAS^®^ HIV DUO AG/AB compared with VIDAS^®^ HIV DUO Ultra. AG antigen, AB antibodies; Table S3: Selected examples of HIV seroconversion panels characterized by viral load and, when available, Fiebig stage, showing detailed AG and AB reactivity profiles obtained with VIDAS^®^ HIV DUO AG/AB, VIDAS^®^ HIV DUO Ultra, and ARCHITECT HIV Ag/Ab Combo assays. AG/Ag antigen, AB/Ab antibodies; Table S4. Detailed results obtained with antibody and antigen performance panels used to compare reactivity profiles between VIDAS^®^ HIV DUO AG/AB and VIDAS^®^ HIV DUO Ultra, including samples representative of early HIV infection stages. AG antigen, AB antibodies.

## Abbreviations

The following abbreviations are used in this manuscript:

CeGIDD	Centres Gratuits d’Information, de Dépistage et de Diagnostic des infections par les virus de l’immunodéficience humaine, des hépatites virales et des infections sexuellement transmissibles
95% CI	95% Confidence Intervals
CLSI	Clinical and Laboratory Standards Institute
CRB	Biological Resource Center
CV	Coefficient of Variation
EFS	French Blood Establishment
ELFA	Enzyme Linked Fluorescent Assay
HCL	Hospices Civils de Lyon
HIV	Human Immunodeficiency Virus
IQR	Interquartile Range
IR	Initially Reactive
QBD	Qualification Biologique du Don
RR	Repeatedly Reactive
LoD	Limit of Detection
ND	Not Determinable
PrEP	Pre-Exposure Prophylaxis
REML	Restricted Maximum Likelihood
SD	Standard Deviation
SPR	Solid Phase Receptacle
S/CO	Signal-to-cutoff
WHO	World Health Organization
