# Supplementary material for: Analytical and Clinical Performance of VIDAS® HIV DUO AG/AB Assay for Enhanced Early HIV Diagnosis
Source: Diagnostics (Basel). 2026 Sep 12;16(18):2954. doi: 10.3390/diagnostics16182954 (PMC13605314; doi:10.3390/diagnostics16182954)
Supplement: Supplementary file 1 [file diagnostics-16-02954-s001.zip › diagnostics-4441793-supplementary.pdf]

Supplementary Materials

Figure S1. Algorithm for HIV infection screening at the Croix-Rousse Hospital (Lyon, France)

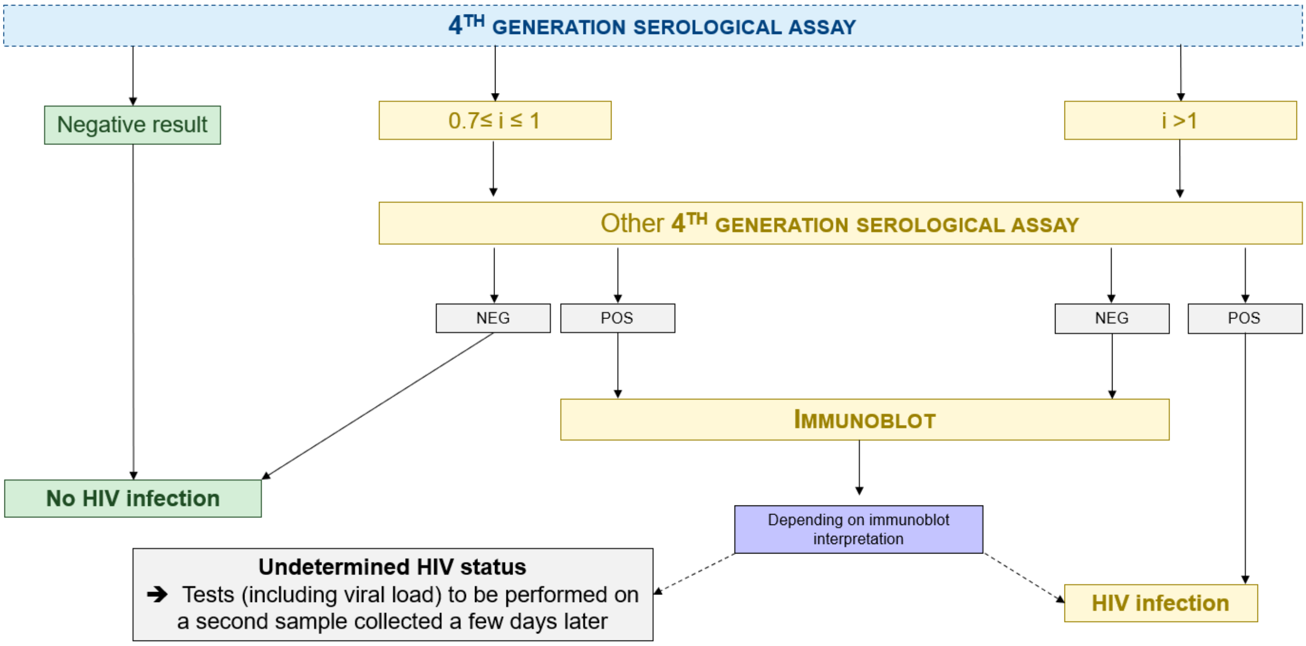

Figure S2. Algorithm for HIV infection screening at the QBD (Qualification Biologique du Don) sites of the French Blood Establishments

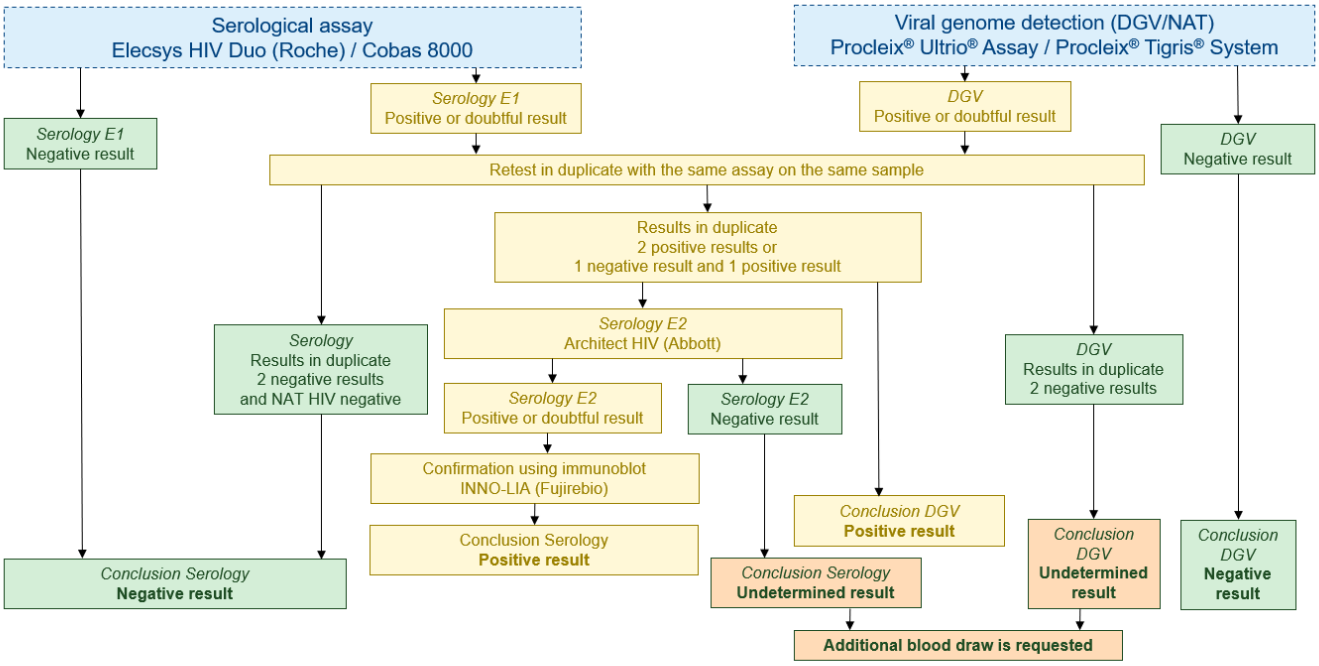

DGV = Détection du Génome Viral
NAT = Nucleic Acid Testing

Table S1. Comparison of the number of bleeds detected with the VIDAS® HIV DUO AG/AB and VIDAS® HIV DUO Ultra assays across 40 HIV seroconversion panels used for early infection sensitivity evaluation. AG/Ag antigen, AB/Ab antibodies

	Number of bleeds detected		
**Panel ID**	**VIDAS® HIV DUO AG/AB**	**VIDAS® HIV DUO Ultra Ag/Ab**	**Difference between VIDAS® HIV DUO AG/AB and HIV DUO Ultra Ag/Ab**
PRB925	2/6	2/6	0
PRB926	4/6	4/6	0
PRB935	2/7	2/7	0
PRB941	4/6	4/6	0
PRB943	5/7	5/7	0
PRB944	5/6	4/6	1
PRB945	4/6	4/6	0
PRB947	3/4	3/4	0
PRB951	4/6	4/6	0
PRB952	4/6	4/6	0
PRB953	3/4	3/4	0
PRB954	3/7	2/7	1
PRB955	4/5	4/5	0
PRB958	4/6	4/6	0
PRB967	4/6	3/6	1
PRB969	5/10	3/10	2
PRB971	3/4	2/4	1
PRB973	3/4	2/4	1
PRB974	3/4	2/4	1
0600-0270	3/4	2/4	1
0600-0271	7/8	6/8	1
0600-0272	3/6	2/6**	1**
HIV6240	7/13	6/13	1
HIV6246	9/21	8/21	1
HIV9011	2/11	2/11	0
HIV9012*	2/6	2/6	0
HIV9013	2/7	2/7	0
HIV9015*	2/6	2/6	0
HIV9017	7/11	4/11	3
HIV9018	4/11	4/11	0
HIV9021	4/17	4/17	0
HIV9022	3/9	3/9	0
HIV9025	2/12	2/12	0
HIV9031	3/19	3/19	0
HIV9032	8/14	7/14	1
HIV9034	3/13	3/13	0
HIV9076*	3/9	3/9	0
HIV9077*	12/23	12/23	0
HIV9079*	7/15	7/15	0
HIV12008	6/13	6/13	0
Total bleeds detected 40 panels	167/358	152/358	17

* 9012 panel samples -05/-07; 9015-01/-02/-07; 9076-07; 9077-13/23/26-29; 9079-14-18 were exhausted, therefore not tested.
** 00-272-04 is AG-positive, -05 back to negative and -06 again AB-positive

Table S2. Representative examples of antibody and antigen reactivity profiles from selected HIV seroconversion panels illustrating earlier or enhanced detection by VIDAS® HIV DUO AG/AB compared with VIDAS® HIV DUO Ultra. AG antigen, AB antibodies

Seroconversion panel		Viral load cp/mL**	Days since 1st bleed	S/CO* VIDAS® HIV DUO AG/AB AB	AG	Interp	VIDAS® HIV DUO Ultra AB	AG	Interp
0600-0272	0600-0272-01	BLD	0	0.22	0.03	NEG	0.24	ND	NEG
	0600-0272-02	BLD	7	0.19	0.01	NEG	0.20	ND	NEG
	0600-0272-03	3,000	11	0.19	0.04	NEG	0.20	ND	NEG
	0600-0272-04	170,000	18	0.52	5.11	POS	0.40	2.04	POS
	0600-0272-05	81,000	25	14.27	ND	POS	0.64	0.80	NEG
	0600-0272-06	74,000	30	12.73	ND	POS	5.40	0.40	POS
HIV9017	HIV9017-01	32,390	0	0.14	1.86	POS	0.36	0.80	NEG
	HIV9017-02	4,516	3	0.11	0.14	NEG	0.24	0.00	NEG
	HIV9017-03	380	7	0.12	ND	NEG	0.24	ND	NEG
	HIV9017-04	404	10	0.15	ND	NEG	0.24	ND	NEG
	HIV9017-05	619	14	0.45	ND	NEG	0.28	ND	NEG
	HIV9017-06	2,040	17	1.12	0.11	POS	0.40	ND	NEG
	HIV9017-07	11,990	21	1.96	0.69	POS	0.52	0.20	NEG
	HIV9017-08	23,590	24	5.65	1.68	POS	1.36	0.64	POS
	HIV9017-09	62,790	28	14.52	ND	POS	2.32	1.84	POS
	HIV9017-10	60,180	32	26.29	ND	POS	4.40	1.68	POS
	HIV9017-11	25,440	35	27.88	ND	POS	4.64	0.88	POS
Total POS samples					10			6	

ND = Not Determinable
*with CO normalized to 1.00 for VIDAS® HIV DUO Ultra
** viral load results from the vendor data sheets

Table S3. Selected examples of HIV seroconversion panels characterized by viral load and, when available, Fiebig stage, showing detailed AG and AB reactivity profiles obtained with VIDAS® HIV DUO AG/AB, VIDAS® HIV DUO Ultra, and ARCHITECT HIV Ag/Ab Combo assays. AG/Ag antigen, AB/Ab antibodies

				S/CO*							
				VIDAS® HIV DUO AG/AB			VIDAS® HIV DUO Ultra			ARCHITECT HIV Ag/Ab Combo**	
**Panel ID**	**Viral load cp/mL*** **	**Day since 1st bleed**	**Fiebig stage**	**AB**	**AG**	**Interp**	**AB**	**AG**	**Interp**	**Ag/Ab**	**Interp**
9018-08	15,280	25	NS	0.37	2.49	POS	0.44	1.32	POS	0.82	NEG
9018-09	193,100	28	NS	ND	24.23	POS	ND	12.84	POS	7.91	POS
9022-06	107	17	NS	0.12	ND	NEG	0.24	ND	NEG	0.14	NEG
9022-07	32,296	23	NS	0.16	2.42	POS	0.32	1.16	POS	0.85	NEG
9022-08	199,644	25	NS	0.52	23.03	POS	ND	11.96	POS	7.94	POS
9022-09	> 500,000	32	NS	ND	82.21	POS	42.76	ND	POS	NS	NS
PRB945-02	700	3	NS	0.24	0.04	NEG	0.24	ND	NEG	0.22	NEG
PRB945-03	90,000	7	NS	0.32	2.90	POS	0.36	1.12	POS	0.73	NEG
PRB945-04	> 800,000	13	NS	ND	33.04	POS	ND	16.00	POS	12.41	POS
PRB945-05	> 800,000	15	NS	ND	40.97	POS	ND	17.80	POS	NS	NS
PRB945-06	> 800,000	20	NS	44.56	ND	POS	21.32	ND	POS	NS	NS
PRB954-04	1,000	10	NS	0.22	0.04	NEG	0.16	ND	NEG	0.20	NEG
PRB954-05	60,000	14	NS	0.26	2.27	POS	0.32	0.88	NEG	0.60	NEG
PRB954-06	600,000	17	NS	0.97	18.97	POS	ND	9.16	POS	4.01	POS
PRB954-07	> 800,000	21	NS	ND	58.54	POS	ND	30.12	POS	NS	NS
PRB969-06	16,000	61	I	0.13	1.04	POS	0.28	0.36	NEG	0.38	NEG
PRB969-07	35,000	63	II	0.16	2.19	POS	0.36	0.96	NEG	0.91	NEG
PRB969-08	110,000	70	IV	13.33	ND	POS	2.28	3.60	POS	18.03	POS
PRB969-09	110,000	72	IV	24.42	ND	POS	4.40	1.84	POS	NS	NS
PRB969-10	210,000	77	V	22.73	ND	POS	5.40	ND	POS	NS	NS
PRB971-01	680	0	I	0.26	0.12	NEG	0.28	0.04	NEG	0.11	NEG
PRB971-02	5,100	2	I	0.31	1.12	POS	0.28	0.44	NEG	0.31	NEG
PRB971-03	320,000	7	II	ND	55.59	POS	ND	32.20	POS	41.70	POS
PRB971-04	2,600,000	11	III	ND	55.53	POS	24.52	ND	POS	NS	NS
PRB974-01	BLD	0	NA	0.14	0.00	NEG	0.24	ND	NEG	0.14	NEG
PRB974-02	11,000	7	I	0.14	1.36	POS	0.32	0.56	NEG	0.41	NEG
PRB974-03	91,000	9	II	0.34	14.32	POS	1.16	5.72	POS	2.98	POS
PRB974-04	920,000	16	II/III	ND	75.66	POS	ND	35.48	POS	NS	NS
Total POS samples					23			18		NA	

Interp = interpretation; ND = Not Determinable; NT = Not tested; NS = not supplied; BLD = below limit of detection; NA = not applicable

* with CO normalized to 1.00 for VIDAS® HIV DUO Ultra

** data from Qiu et al., 2021

*** viral load results from the vendor data sheets

Table S4. Detailed results obtained with antibody and antigen performance panels used to compare reactivity profiles between VIDAS® HIV DUO AG/AB and VIDAS® HIV DUO Ultra, including samples representative of early HIV infection stages. AG antigen, AB antibodies

**Performance panel**		**S/CO***					
		**VIDAS® HIV DUO AG/AB**			**VIDAS® HIV DUO Ultra**		
		**AB**	**AG**	**Interpretation**	**AB**	**AG**	**Interpretation**
EFS test panel DQ121	DQ121-A	3.94	0.09	POS	1.16	0.00	POS
	DQ121-B	100.70	ND	POS	52.36	ND	POS
	DQ121-C	26.12	ND	POS	7.72	ND	POS
	DQ121-D	113.60	ND	POS	64.40	ND	POS
	DQ121-E	85.70	ND	POS	41.80	ND	POS
	DQ121-F	130.16	ND	POS	73.20	ND	POS
	DQ121-G	43.22	ND	POS	15.52	ND	POS
	DQ121-H	109.31	ND	POS	61.32	ND	POS
	DQ121-I	115.10	ND	POS	68.76	ND	POS
	DQ121-J	7.12	0.06	POS	2.04	0.04	POS
	DQ121-K	0.31	0.00	NEG	0.28	0.04	NEG
	DQ121-L	124.74	ND	POS	71.96	ND	POS
	DQ121-M	68.58	ND	POS	27.36	ND	POS
	DQ121-N	123.95	ND	POS	85.64	ND	POS
	DQ121-O	0.30	0.00	NEG	0.20	ND	NEG
	DQ121-P	1.19	0.05	POS	0.44	ND	NEG
	DQ121-Q	2.17	0.04	POS	0.64	ND	NEG
	DQ121-R	93.26	ND	POS	36.12	ND	POS
	DQ121-S	127.01	ND	POS	73.64	ND	POS
	DQ121-T	13.66	ND	POS	3.72	0.00	POS
AccuSet HIV-1 Early infection 0800-0394	0800-0394-08 - Fiebig I	0.29	0.90	NEG	0.28	0.32	NEG
	0800-0394-09 - Fiebig I	0.34	1.62	POS	0.44	0.76	NEG
	0800-0394-12 - Fiebig II	0.50	3.00	POS	0.44	1.32	POS
	0800-0394-13 - Fiebig II	ND	31.64	POS	ND	15.8	POS
	0800-0394-18 - Fiebig IV	5.69	3.88	POS	1.68	1.96	POS
	0800-0394-21 - Fiebig IV	27.44	ND	POS	23.16	ND	POS
	0800-0394-22 - Fiebig V	60.39	ND	POS	32.56	ND	POS
	0800-0394-23 - Fiebig V	32.05	ND	POS	16.28	ND	POS
	0800-0394-24 - Fiebig V	57.77	ND	POS	30.52	ND	POS
	0800-0394-25 - Fiebig V	56.59	ND	POS	24.92	ND	POS
Total POS samples			27			24	

ND = Not Determinable

* with CO normalized to 1.00 for VIDAS® HIV DUO Ultra
